# Supplementary material for: Intravital imaging of real-time endogenous actin dysregulation in proximal and distal tubules at the onset of severe ischemia-reperfusion injury
Source: Sci Rep. 2021 Apr 15;11:8280. doi: 10.1038/s41598-021-87807-6 (PMC8050301; doi:10.1038/s41598-021-87807-6)
Supplement: Supplementary file 1 — Supplementary Legends. [file 41598_2021_87807_MOESM1_ESM.docx]

**SUPPLEMENTARY MATERIAL**

Video 1. Renal tubular filtrative and endocytic capacities impaired by severe ischemia-reperfusion injury. Supplemental Video 1 available at URL: <https://figshare.com/s/9b9624b41b2ef7c0c751>

DOI: 10.6084/m9.figshare.13615889

Video 2. Actin dysregulation at the onset of severe ischemia-reperfusion injury.

Supplemental Video 2 available at URL: <https://figshare.com/s/02d7148b26446224c0e3>

DOI: [10.6084/m9.figshare.14130146](https://doi.org/10.6084/m9.figshare.14130146)
